# Osteoimmune Interactions in Inflammatory Bowel Disease: Central Role of Bone Marrow Th17 TNFα Cells in Osteoclastogenesis

**DOI:** 10.3389/fimmu.2015.00640

**Published:** 2015-12-18

**Authors:** Abdelilah Wakkach, Matthieu Rouleau, Claudine Blin-Wakkach

**Affiliations:** ^1^CNRS, UMR 7370, Laboratoire de PhysioMédecine Moléculaire (LP2M), Faculté de Médecine, Nice, France; ^2^University Nice Sophia Antipolis, Nice, France

**Keywords:** Th17 Cells, osteoclasts, bone marrow, inflammatory bowel diseases, osteoporosis, osteoimmunology

## Abstract

Osteoimmunology is an interdisciplinary research field dedicated to the study of the crosstalk between the immune and bone systems. CD4^+^ T cells are central players in this crosstalk. There is an emerging understanding that CD4^+^ T cells play an important role in the bone marrow (BM) under physiological and pathological conditions and modulate the differentiation of bone-resorbing osteoclasts. However, identification of the mechanisms that maintain CD4^+^ T cells in the BM is still a matter of investigation. This article describes the CD4^+^ T cell populations of the BM and reviews their role as osteoclastogenic population in inflammatory bowel disease.

Bone marrow (BM) has long been known to play an important role in the immune system as a central hematopoietic organ. However, its function in T cell-mediated tolerance or immunity remains elusive. In the past, most of immunological studies were focused on T and B cells in the thymus, the lymph nodes, and the spleen considered as the central reservoirs of adaptive immunity. Over time, it became apparent that the immune system has a far more decentralized governing function. Recently, the BM was shown to play several unexpected roles, serving as an important reservoir for memory T cells including pathogenic cells and long-lived plasma cells involved in the maintenance of long-term immunity and pathogenicity.

## Bone Marrow Resident CD4^+^ T cells

Memory CD4^+^ T cells provide rapid and highly effective protective immunity against previously encountered pathogens and can recognize a wide variety of antigens. The concept of systemic memory consists of two major subsets: central memory T (T_CM_) cells and effector memory T (T_EM_) cells. Importantly, this identification was done in the blood (1). T_CM_ cells express the chemokine receptor CCR7 and the vascular addressing L selectin (CD62L), which enables them to migrate from the blood to the lymph nodes. T_EM_ cells express low levels of CCR7 and CD62L but have receptors that allow them to access peripheral tissues as, for example, the E-selectin ligand cutaneous lymphocyte antigen (CLA), which grants them access to the skin, and α4β7, an integrin that allows them access to the gut. These memory T cells are called tissue-resident memory T cells (T_RM_) (2).

In the BM, T cells represent about 3–8% of total nucleated cells. BM T cells reach the BM from the blood and, after homing to the BM, can move back to the blood to migrate to other lymphoid organs (3). The rules governing cell migration to the BM have been investigated mainly in the case of hematopoietic stem cells (HSCs) and revealed the dominant role of the CXCR4–CXCL12 axis in this migration. Analysis of CD4^+^ T cells from the BM of normal mice or mice affected with inflammatory bowel diseases (IBDs) showed the presence of both T_CM_ and T_EM_ cells that are characterized by a high expression of the chemokine receptors CXCR4 and CCR6 (4, 5). However, the mechanism of recruitment of these T cells in the BM remains elusive. Moreover, the degree to which memory cells are resident (T_RM_) within the BM versus transiting through it is less clear.

In order for T_RM_ cells to be maintained in the BM, they must adapt to the local environment and ignore signals that may induce their egress outside of the BM. The mechanisms and cells involved in the maintenance of BM T cells represent a matter of active research. BM stroma includes mesenchymal stromal cells (MSCs), endothelial cells, osteoblasts (OBLs), and adipocytes. This stromal cell heterogeneity complicates the understanding of the implication of these cells in the maintenance of immunological memory. It is well recognized that BM stromal cells support hematopoiesis by establishing specialized niches. These niches regulate the fate of HSCs in terms of quiescence, migration, and differentiation (6). The major components of the HSC niches include several MSC populations [CXC12-abundant reticular (CAR) cells and Nestin^+^ cells] (7), OBLs (8, 9), and endothelial cells (10, 11). In addition, regulatory T cells (12), macrophages (13), and osteoclasts (OCLs) (14, 15) were shown to contribute to the regulation of the HSC niches. MSCs have also been involved in the retention of T cells in the BM. Tokoyoda et al. showed that memory CD4^+^ T cells are located close to BM stromal cells expressing IL-7 and VCAM1 (16). This was confirmed by Nemoto et al. who reported that in IBD, pathogenic CD4^+^ T cells are retained in the BM through interactions with IL-7-producing MSCs (17). However, the exact nature of the VCAM1^+^ and IL-7^+^ stromal cells and their role in the maintaining of memory CD4^+^ T cells remain to be elucidated. Adoptive transfer of B and T lymphocytes in mice led to seeding of dendritic cell (DC) clusters with grafted cells in perivascular domains, which were referred to as BM immune niches (18). Overall, these observations suggested the existence of CD4^+^ T cell niches that remain to be characterized in term of cell composition and regulation. The identification of BM immune niches raises many new questions. Which molecules regulate T cell migration? How are memory CD4^+^ T cells maintained and for how long? How do they interact with BM stroma under physiological and inflammatory conditions? The identification of BM-specific factors that control T cell functions is likely to have a major impact on translational medicine.

## Crosstalk Between CD4^+^ T cells and Bone Cells

The crosstalk between the immune and bone systems led to the emergence of an interdisciplinary field called osteoimmunology (19). This field emerged from clinical observations reporting that patients presenting an overactivation of the immune system, such as chronic infections and chronic inflammatory diseases, also display bone loss (20–22). The importance of this crosstalk was further confirmed with the identification of the role of activated CD4^+^ T cells in pathological osteoclastogenesis (23). Its scope has been extended to encompass a wide range of molecular and cellular interactions, including those between immune cells and bone cells, and between bone cells and hematopoietic cells. These interactions are essential for the development of the immune and bone systems (15, 24).

Bone remodeling is a highly regulated process involving complex interactions between the activity of the bone-forming OBLs and the activity of the bone-resorbing OCLs. OCLs are multinucleated cells from the myeloid lineage (monocytes and DCs) (25) that degrade the bone matrix and release growth factors that contribute to the coupling between OCLs and OBLs (26). The mesenchymal-derived OBLs migrate to the eroded sites and initiate new bone formation by the secretion of an extracellular matrix and its calcification. In a physiological state, bone remodeling provides an adequate environment for the development of the immune system and the protection of HSCs (6).

The BM represents a reservoir for memory T cells among which 25% are Foxp3^+^ regulatory T cells (27). It is also a preferential site for the migration or the selective retention and function of regulatory T cells. This finding provides evidence for an unidentified role of the BM in T-cell homeostasis. Moreover, Tokoyoda et al. have shown that in adult mice, more than 80% of antigen specific memory CD4^+^ T cells rest in the BM for more than 90 days after immunization and do not proliferate (16). From these observations, we can hypothesize that the immunosuppressive activity of BM regulatory T cells participates in the quiescence of memory T cells as recently shown for CD8^+^ memory T cells (28). In this study, regulatory T cells suppress proliferation and effector programs during the memory differentiation of CD8^+^ T cells in the lymph nodes (28). In addition to regulatory T cells, MSCs are also generally considered as immunosuppressive cells. MSCs may suppress T-lymphocyte proliferation and functions both *in vitro* and *in vivo* (29, 30). They produce soluble factors, including TGF-β, able to mediate suppression of T-cell proliferation (31). MSCs can also inhibit T cell proliferation by increasing IL-10 secretion (31). These properties suggest that the immunosuppressive effect of MSCs may participate to the preservation of CD4^+^ memory T cells in the BM. However, MSCs are a heterogeneous population of cells and their immune suppressive activity has mainly been explored in pathological conditions. It is therefore important to better understand this function to clarify how MSCs may control the quiescence and niche of CD4^+^ T cells.

To date, the role of CD4^+^ regulatory T cells and memory T cells on bone cells in physiological conditions remains controversial. It has been reported that Rag1^−/−^ mice lacking T cells have a normal bone phenotype (32), whereas T cell-deficient nude mice display, with age, an increased bone resorption and a decreased bone mineral density (33). T cells are capable of mediating anti-osteoclastogenic signals, as depletion of CD4 and CD8 T lymphocytes in mice *in vivo* enhances vitamin D3-stimulated OCL formation by blocking the production of osteoprotegerin (OPG) by B cells (34). These observations revealed the difficulty to conclude on the role of CD4^+^ T cells in steady state osteoclastogenesis because they can have direct and indirect effects on OCL precursors and also because of their heterogeneity. Indeed, Th1, Th2, and Th17 cells have been reported to have opposite effects on OCL differentiation *in vitro*. Th1 and Th2 cells inhibit OCL formation through their production of INF-γ and IL-4, respectively, whereas Th17 cells have an osteoclastogenic helper effect *in vitro* mediated by MSCs (35).

## Osteoimmunology Interactions in Inflammatory Bowel Disease

Inflammatory bowel diseases are inflammatory diseases that affect the gastrointestinal tract. There are two main clinical forms of IBD: Crohn’s disease which affects any part of gastrointestinal tract and ulcerative colitis in which the pathology mostly affects the colonic mucosa (36). Several factors including genetic factors, gut microbiota, and the host immune system have been described to contribute to IBD (36). Moreover, these diseases are often associated with extra-intestinal manifestations, in particular, bone loss. Indeed, more than 40% of patients with IBD also present bone loss that remains a major extra-intestinal cause of morbidity leading to an impaired quality of life and productivity (37). The prevalence of osteopenia and osteoporosis in patients presenting with IBD ranges from 22–77% to 17–41%, respectively, depending on the population location or the study design. According to the WHO, osteoporosis is defined as a systemic skeletal disease that occurs when bone resorption exceeds bone formation. In low-turnover osteoporosis, bone resorption is normal whereas the synthesis of bone tissue is reduced. In contrast, in high-turnover osteoporosis, the activity of OCL is increased. Osteoporosis associated with chronic inflammation usually follows the high-turnover pattern, whereas corticosteroid-induced osteoporosis is usually of the low-turnover pattern (38).

The association between chronic inflammation and bone destruction has been recognized a long time ago, leading to the hypothesis of the participation of immune cells in the control of bone remodeling (19). A seminal work published in 1999 by Kong et al. established the role of CD4^+^ T cells in inflammatory osteoclastogenesis. In this study, the authors demonstrated that activated CD4^+^ T cells produce RANKL and induce the differentiation of OCLs *in vitro* (23). Besides that, ctla4^−/−^ mice, in which T cells are spontaneously activated, displayed a severe osteoporosis. Interestingly, transfer of CD4^+^ T cells from ctla4^−/−^ mice into lymphocyte-deficient rag1^−/−^ mice caused bone destruction that can be reversed through inhibition of RANKL by OPG (23). One additional study confirmed these observations and identified a common causal link between intestinal inflammation and bone loss showing that activated T cells producing RANKL accumulate in the BM during intestinal inflammation (39). Taken together, these studies suggest that pathogenic CD4^+^ T cells present in the BM during IBD are potentially osteoclastogenic.

Using a mouse model of colitis, induced in SCID mice by injection of CD4^+^CD45RBhigh naive T cells, Nemoto et al. found that a large number of CD4^+^CD44^+^CD62L^−^ memory T cells resides in the BM. Transfer of BM memory CD4^+^ T cells into new recipient SCID mice reproduced colitis. These BM CD4^+^ cells of colitic mice have therefore been described as “colitogenic memory T cells” (5). Importantly, these resident BM CD4^+^ memory T cells are closely associated with IL-7-producing stromal cells, and they cannot induce colitis when transferred into IL-7^−/−^ × Rag1^−/−^ mice, suggesting that IL-7 plays an essential role in their maintenance or survival in the BM (5). Recently, the same group has demonstrated that BM MSCs are the major source of IL-7 and play a pathological role in IBD by forming the niche for these colitogenic CD4^+^ memory T cells (17). However, their osteoclastogenic effect has not been explored.

Although IBD has traditionally been assumed to be a Th1-dependent disease, there is controversy over the role of Th1 on bone homeostasis. IFN-γ has been shown to directly inhibit osteoclastogenesis by interfering with the RANKL–RANK signaling pathway (40). Moreover, *in vitro* differentiated CD4^+^ Th1 cells have been shown to inhibit OCL formation through their canonical production of IFN-γ (35). In contrast, observations in humans suggested that IFN-γ may promote osteoclastogenesis because it improves bone resorption in osteopetrotic patients treated with IFN-γ (41). IFN-γ is also a physiologic inducer of MHC class II expression by APCs resulting in the activation of T cells that induce bone resorption by their secretion of RANKL and TNF-α (42). Together, these data suggest that IFN-γ inhibits OCL formation through direct targeting of maturing OCLs, while it promotes osteoclastogenesis indirectly by stimulating T cell activation.

Nowadays, it is well known that the inflamed gastrointestinal mucosa of patients with IBD is massively infiltrated with Th17 cells and that Th17-related cytokines are produced in excess (43). Based on this, it is obvious that Th17 cells play an important role in the pathogenesis of IBD, which was previously solely attributed to Th1 cells. However, although a pathogenic role in intestine inflammation has been ascribed to Th17 cells, administration of neutralizing anti-IL-17A antibody to patients with Crohn’s disease did not show any therapeutic benefit (44). Moreover, in some patients, it exacerbated the disease suggesting a protective role of IL-17A (44). Thus, despite the role of Th17 cytokines is important in many aspects of intestinal homeostasis and protection from mucosal pathogens, their role in the pathogenesis of IBD remains ambiguous. However, IL-17 represents a potent osteoclastogenic cytokine, and its receptors are expressed by many cell types, including OBLs and OCLs (45). Kotake et al. reported that IL-17 is abundant in rheumatoid synovial fluid and that IL-17 stimulates OCL differentiation by inducing RANKL expression by OBLs (46). It should be noted that the effect of IL-17 is not limited to this direct action on the OBLs. IL-17 facilitates local inflammation by recruiting and activating immune cells, which leads to an abundance of inflammatory cytokines such as TNF-α (46). IL-17-deficient mice are resistant to bone destruction induced by LPS (35). Otherwise, Oostlander et al. suggested a particular role for IL-17 in osteoclastogenesis in Crohn’s disease patients (47). More recently, we have shown that BM Th17 T cells expressing high levels of TNFα were able to induce OCL differentiation in IBD mice. This T-cell-induced OCL differentiation could be inhibited by IL-17 blockade (4) suggesting the importance of IL-17A in osteoporosis.

A picture emerged from the literature (48, 49) to define what we believe to be osteoclastogenic T cells: first, osteoclastogenic T cells should not produce a large amount of IFN-γ. Second, they should trigger local inflammation and the production of inflammatory cytokines, including TNF-α, that induce RANK-L expression on MSCs. Third, they should express RANK-L and might directly participate in the increase of osteoclastogenesis.

Recent data indicate that in IBD, Th17 cells-producing TNF-α represent the long-sought-after osteoclastogenic T cell subset that fulfills all the criteria mentioned above (4, 50). In murine models of IBD associated with bone loss, we have shown that Th17-producing TNF-α cells accumulate in the BM, the colon, and spleen and have a potent capacity to induce OCL differentiation without addition of any exogenic osteoclastogenic factors (4). Through their production of RANK-L and TNFα, Th17 cells directly induce the differentiation of precursors into OCLs (4), but they also have a major effect on MSCs through their production of IL-17. Indeed, IL-17 increases RANK-L expression in MSCs leading to increased OCL formation (4, 35, 46). Moreover, in the context of IBD, Th17 cells increase the expression of monocyte chemoattractant protein-1 (MCP-1) and macrophage inflammatory protein 1α (MIP-1α) by MSCs, which may promote the recruitment of inflammatory monocytes (OCL precursors) in the BM and their differentiation into OCLs (4). To translate these finding into human disease, IL-17-producing T cells from the blood of IBD patients are osteoclastogenic cells *in vitro* and increase MIP-1α and MCP-1 expression by human MSCs (4, 47), suggesting their participation to osteoporosis in these patients. Therefore, Th17 cells represent a key target for innovative therapeutic approaches for IBD-associated bone destruction.

## Concluding Remarks

Recent advances have contributed to our understanding of the biology of CD4^+^ T cells in the BM. These T cells fulfill both homeostatic and pathological functions with respect to the bone system. IBD is an immune-mediated disease characterized by systemic Th1 and Th17 responses and bone destruction. Recent studies have revealed that Th17 cells are not only required for the initiation of systemic immune response as they are critical regulators in the chronic inflammation associated with bone destruction, particularly in rheumatoid arthritis. Our recent findings extend these observation to IBD in which the site of inflammation is far from the bone and provide the first characterization of osteoclastogenic Th17 TNF-α^+^ cells in the BM linking IBD and bone destruction.

**Figure 1:**
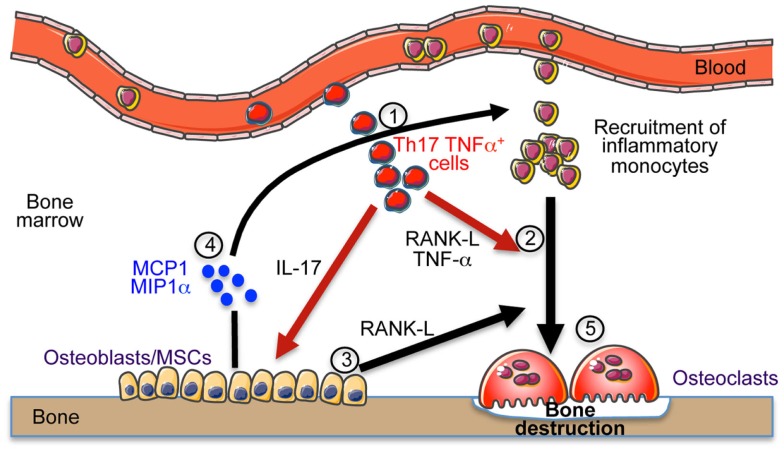
**Th17 cells induce bone destruction in IBD**. In IBD, Th17 TNFα cells migrate and accumulate in the bone marrow (1). They express RANK-L and TNF-α that participate to osteoclast differentiation (2). They also express IL-17 that stimulates the local inflammation and activates osteoblasts to produce RANK-L inducing osteoclast formation (3) and MCP-1 and MIP-1α chemokines (4) participating to the recruitment of osteoclast precursor cells (monocytes) in the BM that contribute to the increased osteoclastogenesis (5) and to bone destruction.

Collectively, we propose that these osteoclastogenic cells, once in the BM, enable the secretion of chemokines and RANKL by stromal cells. This enhances the recruitment of inflammatory monocytes and DCs that differentiate into OCLs and increase the bone resorption leading to osteoporosis as illustrated in Figure 1.

## Author Contributions

AW wrote and reviewed the manuscript. CB-W and MR reviewed the manuscript.

## Conflict of Interest Statement

The authors declare that the research was conducted in the absence of any commercial or financial relationships that could be construed as a potential conflict of interest.

## References

[1] Sallusto F Lenig D Förster R Lipp M Lanzavecchia A. Two subsets of memory T lymphocytes with distinct homing potentials and effector functions. Nature (1999) 401:708–12. 10.1038/44385 10537110

[2] Mueller SN Gebhardt T Carbone FR Heath WR. Memory T cell subsets, migration patterns, and tissue residence. Annu Rev Immunol (2013) 31:137–61. 10.1146/annurev-immunol-032712-095954 23215646

[3] Di Rosa F Pabst R. The bone marrow: a nest for migratory memory T cells. Trends Immunol (2005) 26:360–6. 10.1016/j.it.2005.04.011 15978522

[4] Ciucci T Ibáñez L Boucoiran A Birgy-Barelli E Pène J Abou-Ezzi G Bone marrow Th17 TNFα cells induce osteoclast differentiation, and link bone destruction to IBD. Gut (2015) 64:1072–81. 10.1136/gutjnl-2014-306947 25298539

[5] Nemoto Y Kanai T Makita S Okamoto R Totsuka T Takeda K Bone marrow retaining colitogenic CD4+ T cells may be a pathogenic reservoir for chronic colitis. Gastroenterology (2007) 132:176–89. 10.1053/j.gastro.2006.10.035 17241870

[6] Scadden DT. Nice neighborhood: emerging concepts of the stem cell niche. Cell (2014) 157:41–50. 10.1016/j.cell.2014.02.013 24679525 PMC4161226

[7] Schajnovitz A Itkin T D’Uva G Kalinkovich A Golan K Ludin A CXCL12 secretion by bone marrow stromal cells is dependent on cell contact and mediated by connexin-43 and connexin-45 gap junctions. Nat Immunol (2011) 12:391–8. 10.1038/ni.2017 21441933

[8] Calvi LM Adams GB Weibrecht KW Weber JM Olson DP Knight MC Osteoblastic cells regulate the haematopoietic stem cell niche. Nature (2003) 425:841–6. 10.1038/nature02040 14574413

[9] Zhang J Niu C Ye L Huang H He X Tong W-G Identification of the haematopoietic stem cell niche and control of the niche size. Nature (2003) 425:836–41. 10.1038/nature02041 14574412

[10] Butler JM Nolan DJ Vertes EL Varnum-Finney B Kobayashi H Hooper AT Endothelial cells are essential for the self-renewal and repopulation of Notch-dependent hematopoietic stem cells. Cell Stem Cell (2010) 6:251–64. 10.1016/j.stem.2010.02.001 20207228 PMC2866527

[11] Ding L Saunders TL Enikolopov G Morrison SJ. Endothelial and perivascular cells maintain haematopoietic stem cells. Nature (2012) 481:457–62. 10.1038/nature10783 22281595 PMC3270376

[12] Fujisaki J Wu J Carlson AL Silberstein L Putheti P Larocca R In vivo imaging of Treg cells providing immune privilege to the haematopoietic stem-cell niche. Nature (2011) 474:216–9. 10.1038/nature10160 21654805 PMC3725645

[13] Chow A Lucas D Hidalgo A Méndez-Ferrer S Hashimoto D Scheiermann C Bone marrow CD169+ macrophages promote the retention of hematopoietic stem and progenitor cells in the mesenchymal stem cell niche. J Exp Med (2011) 208:261–71. 10.1084/jem.20101688 21282381 PMC3039855

[14] Kollet O Dar A Shivtiel S Kalinkovich A Lapid K Sztainberg Y Osteoclasts degrade endosteal components and promote mobilization of hematopoietic progenitor cells. Nat Med (2006) 12:657–64. 10.1038/nm1417 16715089

[15] Mansour A Abou-Ezzi G Sitnicka E Jacobsen SEW Wakkach A Blin-Wakkach C. Osteoclasts promote the formation of hematopoietic stem cell niches in the bone marrow. J Exp Med (2012) 209:537–49. 10.1084/jem.20110994 22351931 PMC3302238

[16] Tokoyoda K Zehentmeier S Hegazy AN Albrecht I Grün JR Löhning M Professional memory CD4+ T lymphocytes preferentially reside and rest in the bone marrow. Immunity (2009) 30:721–30. 10.1016/j.immuni.2009.03.015 19427242

[17] Nemoto Y Kanai T Takahara M Oshima S Nakamura T Okamoto R Bone marrow-mesenchymal stem cells are a major source of interleukin-7 and sustain colitis by forming the niche for colitogenic CD4 memory T cells. Gut (2013) 62:1142–52. 10.1136/gutjnl-2012-302029 23144054 PMC3711361

[18] Sapoznikov A Pewzner-Jung Y Kalchenko V Krauthgamer R Shachar I Jung S. Perivascular clusters of dendritic cells provide critical survival signals to B cells in bone marrow niches. Nat Immunol (2008) 9:388–95. 10.1038/ni1571 18311142

[19] Arron JR Choi Y Osteoimmunology: bone versus immune system. Nature (2000) 408:535–6. 10.1038/35046196 11117729

[20] Stellon AJ Davies A Compston J Williams R. Bone loss in autoimmune chronic active hepatitis on maintenance corticosteroid therapy. Gastroenterology (1985) 89:1078–83. 4043665 10.1016/0016-5085(85)90212-4

[21] Feldmann M Brennan FM Maini RN. Role of cytokines in rheumatoid arthritis. Annu Rev Immunol (1996) 14:397–440. 10.1146/annurev.immunol.14.1.397 8717520

[22] Piepkorn B Kann P Forst T Andreas J Pfützner A Beyer J. Bone mineral density and bone metabolism in diabetes mellitus. Horm Metab Res (1997) 29:584–91. 10.1055/s-2007-979106 9479561

[23] Kong Y-Y Feige U Sarosi I Bolon B Tafuri A Morony S Activated T cells regulate bone loss and joint destruction in adjuvant arthritis through osteoprotegerin ligand. Nature (1999) 402:304–9. 10.1038/46303 10580503

[24] Blin-Wakkach C Wakkach A Quincey D Carle GF. Interleukin-7 partially rescues B-lymphopoiesis in osteopetrotic oc/oc mice through the engagement of B220+ CD11b+ progenitors. Exp Hematol (2006) 34:851–9. 10.1016/j.exphem.2006.04.003 16797412

[25] Wakkach A Mansour A Dacquin R Coste E Jurdic P Carle GF Bone marrow microenvironment controls the in vivo differentiation of murine dendritic cells into osteoclasts. Blood (2008) 112:5074–83. 10.1182/blood-2008-01-132787 18768394

[26] Tang Y Wu X Lei W Pang L Wan C Shi Z TGF-beta1-induced migration of bone mesenchymal stem cells couples bone resorption with formation. Nat Med (2009) 15:757–65. 10.1038/nm.1979 19584867 PMC2727637

[27] Zou L Barnett B Safah H Larussa VF Evdemon-Hogan M Mottram P Bone marrow is a reservoir for CD4+CD25+ regulatory T cells that traffic through CXCL12/CXCR4 signals. Cancer Res (2004) 64:8451–5. 10.1158/0008-5472.CAN-04-1987 15548717

[28] Kalia V Penny LA Yuzefpolskiy Y Baumann FM Sarkar S. Quiescence of memory CD8(+) T cells is mediated by regulatory T cells through inhibitory receptor CTLA-4. Immunity (2015) 42:1116–29. 10.1016/j.immuni.2015.05.023 26084026

[29] Nauta AJ Fibbe WE. Immunomodulatory properties of mesenchymal stromal cells. Blood (2007) 110:3499–506. 10.1182/blood-2007-02-069716 17664353

[30] Anderson P Souza-Moreira L Morell M Caro M O’Valle F Gonzalez-Rey E Adipose-derived mesenchymal stromal cells induce immunomodulatory macrophages which protect from experimental colitis and sepsis. Gut (2013) 62:1131–41. 10.1136/gutjnl-2012-302152 22637701

[31] Sioud M. New insights into mesenchymal stromal cell-mediated T-cell suppression through galectins. Scand J Immunol (2011) 73:79–84. 10.1111/j.1365-3083.2010.02491.x 21198747

[32] Anginot A Dacquin R Mazzorana M Jurdic P. Lymphocytes and the Dap12 adaptor are key regulators of osteoclast activation associated with gonadal failure. PLoS One (2007) 2:e585. 10.1371/journal.pone.0000585 17611620 PMC1899087

[33] Li Y Toraldo G Li A Yang X Zhang H Qian W-P B cells and T cells are critical for the preservation of bone homeostasis and attainment of peak bone mass in vivo. Blood (2007) 109:3839–48. 10.1182/blood-2006-07-037994 17202317 PMC1874582

[34] Grcević D Lee SK Marusić A Lorenzo JA. Depletion of CD4 and CD8 T lymphocytes in mice in vivo enhances 1,25-dihydroxyvitamin D3-stimulated osteoclast-like cell formation in vitro by a mechanism that is dependent on prostaglandin synthesis. J Immunol (2000) 165:4231–8. 11035056 10.4049/jimmunol.165.8.4231

[35] Sato K Suematsu A Okamoto K Yamaguchi A Morishita Y Kadono Y Th17 functions as an osteoclastogenic helper T cell subset that links T cell activation and bone destruction. J Exp Med (2006) 203:2673–82. 10.1084/jem.20061775 17088434 PMC2118166

[36] Maloy KJ Powrie F. Intestinal homeostasis and its breakdown in inflammatory bowel disease. Nature (2011) 474:298–306. 10.1038/nature10208 21677746

[37] Tilg H Moschen AR Kaser A Pines A Dotan I. Gut, inflammation and osteoporosis: basic and clinical concepts. Gut (2008) 57:684–94. 10.1136/gut.2006.117382 18408105

[38] Raisz LG. Pathogenesis of osteoporosis: concepts, conflicts, and prospects. J Clin Invest (2005) 115:3318–25. 10.1172/JCI27071 16322775 PMC1297264

[39] Ashcroft AJ Cruickshank SM Croucher PI Perry MJ Rollinson S Lippitt JM Colonic dendritic cells, intestinal inflammation, and T cell-mediated bone destruction are modulated by recombinant osteoprotegerin. Immunity (2003) 19:849–61. 10.1016/S1074-7613(03)00326-1 14670302

[40] Takayanagi H Ogasawara K Hida S Chiba T Murata S Sato K T-cell-mediated regulation of osteoclastogenesis by signalling cross-talk between RANKL and IFN-gamma. Nature (2000) 408:600–5. 10.1038/35046102 11117749

[41] Key LL Rodriguiz RM Willi SM Wright NM Hatcher HC Eyre DR Long-term treatment of osteopetrosis with recombinant human interferon gamma. N Engl J Med (1995) 332:1594–9. 10.1056/NEJM199506153322402 7753137

[42] Gao Y Grassi F Ryan M Pacifici R. IFN-gamma stimulates osteoclast formation and bone loss in vivo via antigen-driven T cell activation. J Clin Invest (2007) 117(1):122–32. 10.1172/JCI30074 17173138 PMC1697800

[43] Ray S De Salvo C Pizarro TT. Central role of IL-17/Th17 immune responses and the gut microbiota in the pathogenesis of intestinal fibrosis. Curr Opin Gastroenterol (2014) 30:531–8. 10.1097/MOG.0000000000000119 25255234 PMC4512208

[44] Hueber W Sands BE Lewitzky S Vandemeulebroecke M Reinisch W Higgins PDR Secukinumab, a human anti-IL-17A monoclonal antibody, for moderate to severe Crohn’s disease: unexpected results of a randomised, double-blind placebo-controlled trial. Gut (2012) 61:1693–700. 10.1136/gutjnl-2011-301668 22595313 PMC4902107

[45] Van bezooijen RL Farih-Sips HC Papapoulos SE Löwik CW. Interleukin-17: a new bone acting cytokine in vitro. J Bone Miner Res (1999) 14:1513–21. 10.1359/jbmr.1999.14.9.1513 10469279

[46] Kotake S Udagawa N Takahashi N Matsuzaki K Itoh K Ishiyama S IL-17 in synovial fluids from patients with rheumatoid arthritis is a potent stimulator of osteoclastogenesis. J Clin Invest (1999) 103:1345–52. 10.1172/JCI5703 10225978 PMC408356

[47] Oostlander AE Everts V Schoenmaker T Bravenboer N van Vliet SJ van Bodegraven AA T cell-mediated increased osteoclast formation from peripheral blood as a mechanism for Crohn’s disease-associated bone loss. J Cell Biochem (2012) 113:260–8. 10.1002/jcb.23352 21898548

[48] Takayanagi H. Osteoimmunology: shared mechanisms and crosstalk between the immune and bone systems. Nat Rev Immunol (2007) 7:292–304. 10.1038/nri2062 17380158

[49] Jones D Glimcher LH Aliprantis AO. Osteoimmunology at the nexus of arthritis, osteoporosis, cancer, and infection. J Clin Invest (2011) 121:2534–42. 10.1172/JCI46262 21737885 PMC3223839

[50] Syrbe U Siegmund B Bone marrow Th17 TNFα cells induce osteoclast differentiation and link bone destruction to IBD. Gut (2015) 64:1011–2. 10.1136/gutjnl-2014-308436 25391836

